# Epigenetic associations in relation to cardiovascular prevention and therapeutics

**DOI:** 10.1186/s13148-016-0170-0

**Published:** 2016-01-15

**Authors:** Susanne Voelter-Mahlknecht

**Affiliations:** University Hospital of Tuebingen, Institute of Occupational and Social Medicine and Health Services Research, Wilhelmstr. 27, 72074 Tuebingen, Germany

**Keywords:** Cardiovascular disease, Epigenetics, Therapy, Prevention

## Abstract

Cardiovascular diseases (CVD) increasingly burden societies with vast financial and health care problems. Therefore, the importance of improving preventive and therapeutic measures against cardiovascular diseases is continually growing. To accomplish such improvements, research must focus particularly on understanding the underlying mechanisms of such diseases, as in the field of epigenetics, and pay more attention to strengthening primary prevention.

To date, preliminary research has found a connection between DNA methylation, histone modifications, RNA-based mechanisms and the development of CVD like atherosclerosis, cardiac hypertrophy, myocardial infarction, and heart failure. Several therapeutic agents based on the findings of such research projects are currently being tested for use in clinical practice. Although these tests have produced promising data so far, no epigenetically active agents or drugs targeting histone acetylation and/or methylation have actually entered clinical trials for CVDs, nor have they been approved by the FDA. To ensure the most effective prevention and treatment possible, further studies are required to understand the complex relationship between epigenetic regulation and the development of CVD. Similarly, several classes of RNA therapeutics are currently under development. The use of miRNAs and their targets as diagnostic or prognostic markers for CVDs is promising, but has not yet been realized. Further studies are necessary to improve our understanding of the involvement of lncRNA in regulating gene expression changes underlying heart failure. Through the data obtained from such studies, specific therapeutic strategies to avoid heart failure based on interference with incRNA pathways could be developed.

Together, research and testing findings raise hope for enhancing the therapeutic armamentarium. This review presents the currently available data concerning epigenetic mechanisms and compounds involved in cardiovascular diseases, as well as preventive and therapeutic approaches against them.

## Background

In the western world, cardiovascular disease is the most common cause of human morbidity and mortality. Expenses for the treatment of cardiovascular disease to the European health care system are presumed to be as high as 200 billion Euros each year [1].

In the USA, the overall rate of deaths attributed to cardiovascular disease (CVD) in 2011 was 229.6 per 100,000 Americans [2]. Thus, CVD still accounted for 31.3 % (786 641) of all 2,515,458 deaths in the USA [2]. Based on the death rate data from 2011, more than 2150 Americans die of CVD each day, which equals an average of one death every 40 s [2]. The cardiovascular disease epidemic is rapidly spreading throughout the world. There are estimates that in 2030, nearly 23.6 million people will die from CVD worldwide [3, 4]. Due to an aging population and shifting risks posed by the environment, this burden is expected to increase in developing countries. In these countries, diets are changing to include higher sodium and fat content. The majority of the fatalities caused by CVD are, however, preventable [5, 6]. According to epidemiological and clinical studies, lifestyle modifications to nutrition and exercise can be initial protective measures to reduce CVD risk [7, 8].

In addition, mortality rates are higher and prognoses are generally worse in patients with diabetes suffering a cardiovascular event [9]. As a consequence of better primary and secondary prevention, there is a decrease in cardiovascular events in type 1 and type 2 diabetic patients, as well as an increased life expectancy. Nevertheless, epidemic bursts of obesity in connection with decreased physical activity and increased survival in the general population have also led to a higher incidence of type 2 diabetes worldwide. This in turn contravenes the decrease in diabetes-related mortality that would otherwise increase significantly in the coming decades [9].

Despite the improvement of cardiovascular outcome and survival of heart failure patients through strategies of classical pharmacological treatment (e.g., the use of beta-blockers and angiotensin-converting enzyme (ACE) inhibitors), such therapies are ultimately unable to prevent further progression of the disease itself [10]. Thus, a more thorough understanding of underlying mechanisms, in the field of epigenetics, for instance, and the development of innovative and more effective therapies for heart diseases is necessary.

## Review

Epigenetic alterations in gene expression may be achieved through changes in the tertiary structure of a DNA strand. Without altering the DNA sequence itself, epigenetic alterations in the form of chromatin-based modifications affect only the expression of the targeted genes [11]. Among these modifications are the methylation of DNA, the posttranslational modification of histone proteins, and RNA-based mechanisms [12]. The development of therapeutic strategies has been strongly encouraged by the reversible nature of such epigenetic alterations, as they allow direct targeting by various epigenetic components [11].

Preliminary research has shed light on the correlations between DNA methylation, histone modifications, and RNA-based mechanisms with CVD including atherosclerosis, heart failure, myocardial infarction, and cardiac hypertrophy. Currently, several therapeutic agents based on these mechanisms are being tested for their potential utility in clinical practice [13]. Although much data have been accumulated so far, no epigenetically active agents have entered clinical trials for CVD. Research on the potential use of epigenetically active compounds to treat these pathologies is, thus, only preliminary.

**Fig. 1:**
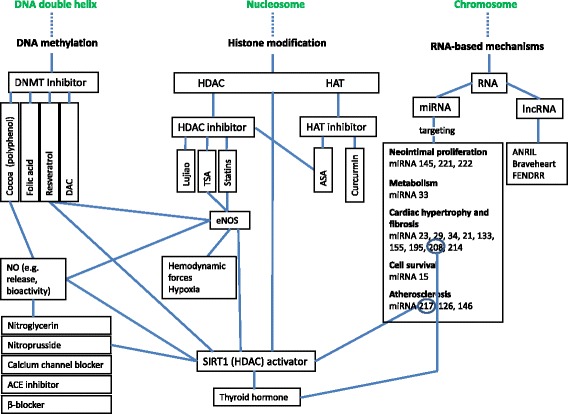
Potential epigenetic mechanisms (DNA methylation, histone alteration, RNA-based mechanisms) and compounds involved in cardiovascular disease. *ACE* angiotensin-converting enzyme, *ANRIL* anti-sense non-coding RNA, *ANRIL* anti-sense non-coding RNA in the INK4 locus, *ASA* acetylsalicylic acid, *ASOs* anti-sense oligonucleotides, *CHD* coronary heart disease, *CVD* cardiovascular disease, *DAC* 5-aza-2-deoxycytidinedemethylating agent, *DNMT* DNA methyltransferase, *eNOS* endothelial nitric oxide synthase, *FENDRR* FOXF1 adjacent non-coding developmental regulatory RNA, *HAT* histone acetyltransferase, *HDAC* histone deacetylase, *lncRNA* long non-coding RNA, *miRNA* microRNA, *NO* nitric oxide

In the following, the currently available data concerning epigenetic mechanisms and compounds involved in cardiovascular diseases will be presented. The assignment of the discussed substances to different mechanisms of action is shown in Fig. 1. It has to be differentiated between the mechanisms of DNA methylation, histone modification, and RNA-based mechanisms.

### DNA methylation (Fig. 1)

The methylation of DNA is regulated by DNA methyltransferases (DNMT1, DNMT3a, and DNMT3b) in the presence of S-adenosyl-methionine; this is the methyl donor for methylation of cytosine residues at the C-5 position to yield 5-methylcytosine [14, 15]. DNA methylation states regulate biological processes underlying CVD, such as atherosclerosis, inflammation, hypertension, and diabetes [16–19].

One DNA methyltransferase (*DNMT*) *inhibitor* is 5-aza-2-deoxycytidinedemethylating agent (*DAC*), which induces the reexpression of hypermethylated silenced genes which might include the reexpression of hypermethylated estrogen receptor (ER) alpha and ER beta in normal smooth muscle and endothelial cells. The failure of estrogen therapy to exert cardioprotective effects might be explained by the silencing of ERs in women that result from epigenetic changes. Thus, CVD may be prevented successfully through the combined use of epigenetic and hormone replacement therapy [13].

Studies in humans have shown that certain dietary compounds can modulate the status of DNA methylation [13]. As one of the largest and most ubiquitous groups of phytochemicals, *polyphenols* are contained in fruits, vegetables, and other dietary components including green tea, red wine, and cocoa [20, 21]. Various studies have indicated a connection between a polyphenol-rich diet and a reduced risk of CVD [13, 22–24]. Polyphenols are believed to be the principal anti-inflammatory mediators. Inflammation is a cardiovascular risk factor in and of itself, while all other cardiovascular risk factors can, in turn, be linked back to inflammation [25, 26].

One of the main sources of polyphenols in the human diet are *cocoa* products. The protective capacity of these cocoa polyphenols in connection with CVD inflammation has been the target of many human intervention studies [27].

Cocoa polyphenols possess a range of cardiovascular protective properties and can play a meaningful role by modulating different inflammatory markers involved in atherosclerosis [27]. Numerous population studies found a correlation between cocoa intake (e.g., chocolate) and reduced CVD risk [13], as well as an inverse association between cocoa intake (e.g., chocolate) and CVD mortality [22, 28–30]. In the study by Janszky [29], however, the occurrence of uncontrolled confounders could not be excluded. Furthermore, the patients were only asked about their general chocolate consumption without a differentiation between dark and milk chocolate.

Substantial evidence suggests that the consumption of cocoa has an effect on multiple cardiovascular risk factors, such as blood pressure [31], lipid profiles [32], and flow-mediated vascular dilatation [27, 33]. Nevertheless, analytical works like that by Jia and colleagues also have their limitations [32]. For one, the quality of the studies included in their meta-analysis varies significantly: Based on the standard for clinical trials of prescribed medicine, only three of the eight trials discussed were high-quality studies (Jadad score ≥4), while the other five studies were of low quality. This suggests that more high-quality, large, randomized, and double-blinded studies are necessary to secure data on this issue. Additionally, the effectiveness of long-term cocoa supplementation on the lipid profile could not be supported by any reports. While human and animal experiments show that the effect of cocoa is indeed dependent on pretreatment concentrations of cholesterol, to date, the effect of cocoa in dyslipidemia patients has not been tested in randomized trials. These studies only tested soft endpoints, meaning cholesterol changes from baseline and not clinical outcomes of the treatment.

The report by Hooper et al. [34], on the other hand, is the first systematic assessment of the effectiveness of the range of flavonoid subclasses and flavonoid-rich food sources on CVD risk factors based on randomized controlled trials [34]. Nevertheless, the review lacks quantifying approaches to the effects of flavonoid-rich food and extracts on CVD risk factors, as it does not include studies assessing their effects on CVD. In addition, it fails to provide sufficient well-designed studies of risk factors for most flavonoids. The small number and size of the included studies, as well as significant differences of the baseline levels of specific outcomes between intervention and control arms in parallel studies, the missing report of a paired *t* test in crossover studies, and a variety of studies based on missing or poorly reported data present further weaknesses of the review. The methodological validity of the review is similarly problematic, mostly due to its lack of evidence concerning adequate allocation concealment, its blinding procedures, the similarity of intervention and control arms in terms of saturated fat intake and the predominance of industrially funded studies, as well as possible reporting bias. Altogether, this may lead to an exaggerated image of effectiveness [35]. Where Hooper et al. present evidence on the effectiveness of flavonoid-rich food, it is unclear whether the flavonoids themselves solely or partially cause the observable effects, or whether other bioactive components are responsible for the same [34].

Cocoa extract has been found to inhibit the expression levels of genes encoding DNMTs and methylenetetrahydrofolate reductase (MTHFR) in vitro [20]. Polymorphisms in the MTHFR gene have been found to be possible risk factors for a variety of common conditions, e.g., heart disease.

Cocoa polyphenols decrease the susceptibility of low-density lipoprotein (LDL) to oxidation and inhibition of platelet activation and aggregation [36–40]. In addition, cocoa polyphenols affect the most significant markers of CVD: lipid profiles [36, 41], blood pressure [31, 42, 43], hemostasis [44–46], and endothelial dysfunction [47–49].

The study by Faridi et al. [49] is the first to examine how sugar-free cocoa acutely affects endothelial function and blood pressure, but it also has its limitations. For one, the findings apply to acute cocoa ingestion only. The measurements of endothelial function are limited to a single time point, which makes them prone to neglect temporal and maximal differences. In addition, since plasma catechin concentrations were not measured, it is unclear whether effects could be attributable to these components or not. Moreover it remains uncertain whether the observed effects were exclusively caused by the deliberate interventions, as the study lacked in dietary information and control of its subjects. The homogeneous population of the study, consisting mainly of white women concentrated around the Naugatuck Valley, finally allows only limited generalizations of its findings.

It is well known that alterations in plasma cholesterol levels (LDL-c and high-density lipoprotein (HDL-c)) are related to the progression of atherosclerosis and CVD [50]. Studies have shown dietary interventions with cocoa powder in mild hypercholesterolemic patients to significantly lower levels of LDL-c [51], while the level of HDL-c was increased in normo- and mild hypercholesterolemic patients after consuming dark chocolate or cocoa powder [37, 51, 52].

In addition, cocoa polyphenols have an effect on nitric oxide (NO) [27, 35, 47]. After consuming cocoa beverages which contained different contents of flavanols, healthy subjects exhibited an increase in their plasma nitric oxide.

*Folic acid and B vitamins*, *other kinds of DNMT inhibitors*, directly supply the one-carbon-metabolism with methionine for the production of S-adenosylmethionine [53]. A deficiency in folic acid leads to global DNA hypomethylation which correlates with an increased risk of cancer and cardiovascular diseases, such as atherosclerosis, coronary heart disease (CHD), and anemia [54–56].

Yang et al. [56] tried to avoid limitations of meta-analyses, like search comprehension and selection, the accuracy of study methodologies, and publication bias, by maximizing study identification and minimizing biases through an a priori development of the study protocol, a profound survey of several databases, and the definition and execution of explicit criteria for the selection of applicable studies, data collection, and data analysis. As a positive result, the results of the included trials did not exhibit any substantial heterogenities.

Vitamin B complex (folic acid and vitamins B6 and B12) substitution therapy leads to a decreased plasma homocysteine level [57]. Folic acids themselves are necessary to methylate homocysteine (Hcy) to methionine. Low serum levels of folic acid have furthermore been connected to increased serum levels of Hcy [13]. Hcy serum levels are increased through the common C677T variant in MTHFR. The risk of CVD increases 14–21 % in individuals with the homozygous TT genotype [58]. Daily dietary supplementation with folic acid and B vitamins reduces plasma Hcy levels, which in turn decreases the risk of CVD in healthy subjects or improves the likeliness of survival in patients with CHD [59]. This is, nevertheless, a controversial effect, as the reducing Hcy concentrations has not proven to be beneficial in the majority of clinical studies. Although folic acid supplementation shows a potentially modest benefit in stroke prevention, it does not seem to exhibit benefits in connection with preventing CHD and CVD [56, 60]. On the contrary, a meta-analysis indicated a potential detrimental effect of folic acid in subjects with high baseline Hcy [60, 61]. However, the study by Miller et al. [60] is limited by (i) differences in the composite clinical end points between trials and in CVD risk factors for trial participant-based eligibility criteria; (ii) an inconsistent and incomplete discussion of baseline homocysteine level results that preclude a more detailed assessment of interactions; and (iii) neglecting that subgroup effects and potential interactions may occur solely by chance.

Homocysteine is a serious, independent risk factor for atherosclerosis [57]. Vascular complications and an increased cardiovascular risk in connection with increased circulation levels of homocysteine may, as various studies indicate, be caused by the methylation of DNA [62]. It is assumed that lowering plasma homocysteine levels will result in a decreased risk of CVD [13].

Nevertheless, recent large randomized studies (VISP, NORVIT, and HOPE-2) [63, 64] could not conclude that this is actually the case. These studies, particularly “NORVIT” and “VISP,” have specific deficiencies: The “NORVIT” study did not include vitamin deficiency and increased homocysteine levels as inclusive criteria. Although a preventive effective of vitamins can only be expected after 3 years [65], this study also considered secondary findings that were observed immediately after a cardiac event. Folic acid levels in the placebo group of this study, for instance, rose from an initial 9.6 to 13.1 nmol/l, similar to the verum group. This suggests that over time, the placebo group may have become nonexistent. Furthermore, Walk et al. suggest that a statistically sound evaluation of the effects of decreasing homocysteine levels through secondary prevention would have required the observation of ca. 20,000 patients over a period of 5 years [65]. The “VISP” study, on the other hand, only used high-dose and a low-dose vitamin groups, but no placebo group [66]. Incidentally, in 1998, the USA and Canada passed a law on the enrichment of wheat products with folic acid, which would have falsified and thus annihilated the further examination of folic acid deficiencies in test subjects [66]. Moreover, Rajan et al. concluded from a study published in 2002 [67] that their high-dose group was still not receiving enough vitamin B12 to compensate an insufficient gastrointestinal resorption of the vitamin, which led to a further blurring of the results between the low-dose and high-dose groups.

The polyphenol *resveratrol* (*RESV*) is a *DNMT inhibitor* found in various plants, including grapes, berries and peanuts [68], and in processed foods such as red wine [69]. It modulates the expression of several targets, such as sirtuin 1, p38 mitogen-activated protein kinase (MAP38 kinase), nuclear factor “kappa-light-chain-enhancer” of activated B cells (NF-κB), activating protein-1 (AP-1), endothelial nitric oxide synthase (eNOS), as well as inflammatory cytokines, like tumor necrosis factor alpha and interleukin-6 and interleukin-8, as well as vascular cell adhesion molecule-1 (VCAM-1) and intercellular adhesion molecule-1 (ICAM-1) [13, 70]. Metabolic disorders, chronic heart disease, and inflammatory diseases can be improved by resveratrol through its upregulation of SIRT1 in endothelial cells [70]. Resveratrol also possesses a number of bioactivities, such as antioxidant, anti-inflammatory, and cardioprotective effects [69].

In vitro, RESV has exhibited the ability to enhance the activity and expression of eNOS [68]. It was also found to prevent an increase in vasoconstrictors, like angiotensin II and endothelin-1, as well as intracellular calcium, in mesangial cells [69]. Antioxidative effects of resveratrol on low-density lipoproteins (LDL) were identified by Frankel et al. in 1993 [71].

In conclusion, unaltered epigenetic regulation pathways like DNA methylation and posttranslational chromatin modifications constitute the basis of a healthy cardiovascular system. To date, many studies have shown the possibilities and positive effects of treatment with epigenetic modulators.

### Histone modification (Fig. 1)

1. Histone acetyltransferase (HAT) modification

The HAT inhibitor *curcumin* (diferuloylmethane) is a polyphenol present in a curry spice exhibiting a diverse range of molecular targets, such as growth factors and their receptors, transcription factors, enzymes, cytokines, and genes regulating cell proliferation and apoptosis [13].

Curcurmin modulates epigenetic factors [72] and has effects on the regulation of histone deacetylases, histone acetyltransferases, DNA methyltransferase I, and miRNAs [72]: Out of the various studies that have been conducted on the effects of curcumin on histone deacetylase (HDAC) expression, Bora-Tatar et al. [73] found that from among 33 carboxylic acid derivatives, curcumin proved the most effective HDAC inhibitor and, as such, even more potent than the well-known HDAC8 inhibitors valproic acid and sodium butyrate. HDAC 1, 3, and 8 protein levels can be significantly decreased by curcumin [73–76].

To the contrary, HDAC2 was activated, restored, and increased in its protein expression by curcumin [77, 78].

Obviously, the different effects of curcumin on the various subtypes of HDAC enzymes indicate the necessity of further research to secure an understanding of the exact relationship between curcumin and HDAC expression [72].

Curcumin is furthermore a potent HAT inhibitor. It inhibits p300 [79–86], inhibits GCN5 associated with hypoacetylation of histone H3 [87], and inhibits GCN5 associated with hypoacetylation of histone H3 [87].

The fact that curcumin modulates both HDAC and HAT suggests a common underlying mechanism. Oxidative stress can, for instance, activate NF-κB through the stimulation of intrinsic HAT activity, which results in the expression of pro-inflammatory mediators, but it can also inhibit HDAC activity [88]. Curcumin as an antioxidant may similarly influence both acetylation and deacetylation by regulating oxidative stress [72].

Nevertheless, only a few studies have investigated the effect of curcumin on the methylation of DNA [72]. For one, it seems to covalently block the catalytic thiolate of DNMT1 in order to exert its inhibitory effect on DNA methylation [89]. This previous understanding was however thwarted by a more recent study that showed no curcumin-dependent demethylation, which in turn suggests that curcumin has little or no pharmacological relevance as a DNMT inhibitor [90]. These contradictions and other inconsistencies about the influence of curcumin on DNMT point to the urgency of further research in this area [72]. Additionally, curcurmin is known to covalently block the catalytic thiolate of C1226 of DNA methyltransferase I [89] and to induce global genomic DNA hypomethylation [89].

Further, Sun et al. [91] found that curcumin altered miRNA expression in human pancreatic cancer cells, in which miRNA-186 and miRNA-199a were the most downregulated and miRNA-22 was the most upregulated [72, 92]. This curcumin-induced upregulation simultaneously suppressed the expression of its target genes Sp1 and estrogen receptor 1 [91].

Curcumin also downregulates the activation of nuclear factor kappa-light-chain-enhancer of activated B cells (NF-κB) by various tumor promoters, including phorbol ester, tumor necrosis factor, and hydrogen peroxide [93, 94]. Curcumin-induced downregulation of NF-κB is mediated through the suppression of the activation of IκBα kinase (IKK) [93].

Protective effects of curcurmin on the cardiovascular system have been demonstrated [95]. In fact, the administration of curcumin to healthy volunteers and patients with atherosclerosis has proven to significantly lower LDL levels and increase HDL levels [96, 97]. Both a rat model of heart failure and primary cultured rat cardiac myocytes and fibroblasts have shown curcumin to prevent ventricular hypertrophy and to perpetuate systolic function [98]. In rat cardiomyocytes, curcumin seems to act through two or more mechanisms at once: through the inhibition of histone acetylation and hypertrophy-responsive transcription factors, including GATA binding protein 4 (GATA4), as well as through the disruption of p300/GATA4 complex [99].

In human subjects, studies indicated that *acetylsalicylic acid* (ASA) therapy reduces ABCA1 DNA methylation levels that are independent from aging and CHD status of patients, which suggests that this molecular mechanism is involved in the pathophysiology of CHD and thus points towards new therapeutic strategies [100].

2. HDAC modification

The *sirtuin 1 protein* (*SIRT1*) is a member of the class III NAD+-dependent histone deacetylases. These sirtuins, and particularly SIRT1, participate in the response to DNA damage, metabolism, longevity, and carcinogenesis. Furthermore, different cellular processes, such as differentiation, proliferation, and apoptosis through deacetylation of important regulatory proteins such as p53, FOXO3a, and NF-κB, are regulated by SIRT1.

The activity and expression of human SIRT1 can be activated or inhibited by various modifiers, including food and cosmetic additives. Agents such as l-thyroxin and sodium nitroprusside frequently used in clinical practice were identified to be potent activators of human SIRT1 expression [101]. The exposure of euchromatin-associated epigenetic marks to T3 induces SIRT1 by enhancing histone acetylation and RNAP II recruitment [102].

Furthermore, the treatment of peripheral blood mononuclear cells (PBMCs) with sodium nitroprusside has been associated with a high increase in cellular lifespan, while l-thyroxin was unable to prolong lifespan, which suggests that an isolated upregulation of SIRT1 is insufficient to promote longevity [101]. Perhaps these agents can be used in everyday clinical practice for indications with a tolerable number of side effects [103–106].

*Thyroid hormones* can contribute to cardiac repair and regeneration through the reactivation of developmental gene programming [107] and can have important effects on heart remodeling through mir-208 [108]. Thyroid hormones have been identified as independent determinants of functional recovery and mortality after myocardial infarction [107]. Concerning the potential for therapeutic measures, thyroid hormones as an agent contributing to the regeneration or repair of the ischemic myocardium are awaiting testing in clinical trials [109].

*Sodium nitroprusside* is a medical agent that belongs to the class of cyanides. It is highly efficient in decreasing blood pressure and exerts vasodilating activity through release of *nitric oxide* (*NO*) in smooth muscle cells and the vessel wall. NO is thus a vasodilator that mediates multiple physiological and pathophysiological activities in the cardiovascular system and protects against the development of atherosclerosis [13].

The signaling molecule nitric oxide is produced in endothelial cells by eNOS [110]. eNOS, which is encoded by the gene NOS3, catalyzes the generation of NO from L-arginine in blood vessels [111]. NOS3 is the best-characterized endothelial gene in association with cardiovascular physiology [13]. Endothelial dysfunction is primarily characterized by a reduction in eNOS expression and the bioavailability of NO [112]. This reduction, which takes place in the neointimal coverings of advanced atheromatous plaques, is a prominent feature of endothelial dysfunction [113–116].

A variety of risk factors contributes to dysfunction of endothelial cells through changes in eNOS expression and activity [113–116]: Both transcriptional and post-transcriptional mechanisms controlling eNOS mRNA levels have been associated with the same [117–120]. The differential expression of eNOS is, furthermore, caused by such hemodynamic forces at characteristic regions within the vasculature as arterial curvatures and bifurcations [113].

Additionally, hypoxia is known to significantly downregulate NOS gene expression in endothelial cells [13, 121] in association with a post-transcriptional downregulation of eNOS mRNA expression [122, 123]. Post-transcriptional regulation of eNOS mRNA stability is an important component of eNOS regulation, especially under hypoxic conditions [110].

The expression of eNOS is induced and directly upregulated by SIRT1 [124]. SIRT1 may also be related to the inhibition of oxidized low-density lipoprotein (oxLDL)-induced apoptosis and the improvement of endothelial relaxation [124]. Due to these mechanisms, SIRT1 is considered an anti-atherosclerotic factor [124]. The activation of SIRT1 is thus an important innovative therapeutic target in future treatment strategies of cardiovascular disease [125, 126].

*NO insufficiency*—e.g., in atherosclerosis, hypertension, arterial thrombotic disorders, heart failure, coronary heart disease, and stroke [11, 17, 127–134]—may reflect an absolute deficit of NO (synthesis), impaired availability of bioactive NO, or enhanced NO inactivation. A broad spectrum of pharmacotherapeutics in cardiovascular medicine works on either the replacement or augmentation of endogenous NO through exogenously administered NO donors.

For decades, clinical practice has used several *NO donors* (e.g., *nitroglycerin* and *nitroprusside*), for instance to treat hypertension and heart failure [135]. Due to their therapeutic half-life, systemic absorption with potentially adverse hemodynamic effects, and drug tolerance, conventional nitrate compounds are limited in their treatment applications [17, 127]. Novel NO donors offering selective effects, a prolonged half-life, and a diminished incidence of drug tolerance have been developed to overcome these limitations.

*Calcium channel blockers*, e.g., dihydropyridine calcium channel antagonists, have been a common treatment of angina pectoris and hypertension for years [136]. They act through inhibiting the smooth muscle L-type calcium current, which decreases the intracellular calcium concentration and induces smooth muscle relaxation. Dihydropyridine can, furthermore, initiate the release of NO from the vascular endothelium [136].

*Angiotensin-converting enzyme* (ACE) *inhibitors* are cardiovascular agents that modulate *endogenous NO bioactivity*. ACE degrades bradykinin [137] and generates angiotensin II; bradykinin, in turn, has exhibited the ability to stimulate the endothelium to release vasodilating substances, in particular NO. By increasing bradykinin, ACE inhibitors may thus enhance the release of endothelial NO. It has been shown that ACE inhibitors exert some of their beneficial pharmacological effects by increasing vascular NO activity [138–140].

*β-blockers* may also obstruct the NO pathway [135]. Nebivolol, a β_1_-blocker and chemical racemate containing equal proportions of d- and l-enantiomers [141], for instance, reportedly induces endothelium-dependent arterial relaxation in dogs in a dose-dependent manner [142]. The endothelium-dependent relaxation caused by nebivolol is abolished by *N*-nitro-l-arginine methyl ester, an inhibitor of NO synthase [135].

Dysfunction of histone acetylation has been associated with the pathogenesis of chronic heart failure. *Lujiao*, an agent that is long known in traditional Chinese medicine, has previously been used in the treatment of heart failure [143]. This medicine has been considered a potential therapy for hypertrophic cardiomyocytes on histone acetylation [143].

Histone deacetylase (HDAC) “inhibitors attenuate pathological cardiac remodeling and hypertrophic gene expression; yet, the direct histone targets remain poorly characterized” [144]. There are studies suggesting “a mechanism for cardioprotection subject to histone deacetylation as a previously unknown target, implicating the importance of inflammation by pharmacological HDAC inhibition” [144].

HDAC inhibitor *trichostatin A* (TSA) is an anti-fungal antibiotic agent that is being produced by Streptomyces platensis that selectively blocks class I and class II HDACs in mammals, but not class III HDACs [145]. TSA is known to be an epigenetic modulator [146] and HDAC inhibitor [146]. Although TSA decreases both eNOS protein and mRNA levels, TSA paradoxically enhances the activity of the eNOS promoter and does not alter the eNOS transcription rate in nuclear run-on experiments. This suggests that TSA post-transcriptionally targets eNOS mRNA. eNOS expression in ECs seems to be regulated partly by HDAC-dependent mechanisms [147]. Furthermore, trichostatin A treatment increases the expression of (encoding estrogen receptors) ERa and ERb in endothelial cells [13]. Encoding estrogen receptors ERa and ERb (ESR1 and ESR2, respectively), atheroprotective genes are consistently hypermethylated in human coronary atherosclerotic tissues and plaque regions of the ascending aorta [13]. Both smooth muscle cells and endothelial cells exhibit ERs in the coronary arterial wall which may protect against atherosclerosis, especially in CHD. Deficiencies in ERa have proven to accelerate atherosclerosis in human subjects [133]. The risk of cardiovascular diseases can be reduced about 30 to 50 % by estrogen substitution [148].

In vivo studies in animals have shown treatment with trichostatin A to improve functional myocardial recovery after myocardial infarction. Treatment of myocardial infarction with trichostatin A has proven to increase angiogenesis [13]. It can hence be concluded that HDAC inhibition may lead to the preservation of cardiac performance and mitigate myocardial remodeling through the stimulation of endogenous cardiac regeneration [149]. HDAC inhibition has furthermore been shown to enhance the formation of myocytes and microvessels in the heart. Due to its ability to stimulate angiogenesis, HDAC inhibition is capable of minimizing the loss of myocardial performance following myocardial infarction [13].

*Statins* are used as primary and secondary prophylaxis against atherosclerosis and cardiovascular incidents [150]. They also act as a first-line treatment to decrease serum cholesterol levels in patients with high cholesterol [13] (e.g., polygenetic hypercholesterolemia or familial hypercholesterolemia but also combined hyperlipidemia) by inhibiting 3-hydroxy-3-methylglutaryl coenzyme A (HMG CoA) reductase [135].

In addition, statins have many pleiotropic effects. Among these are beneficial effects on endothelial function and blood flow, decreased LDL oxidation, enhanced atherosclerotic plaque stability and decreased proliferation of vascular smooth muscle cells and platelet aggregation, as well as reduced vascular and atherosclerotic inflammation, which is caused by histone modifications and an inhibited release of pro-inflammatory cytokines [13].

They may furthermore possess antioxidant properties [151] and be able to upregulate eNOS activity and expression (via the inhibition of Rho) [135]. Through the induction of hypoxia and oxidized LDL, statins reverse the downregulation of eNOS expression [152], which may ultimately be the reason for their ability to improve the vascular bioactivity of NO [153] and atherosclerotic plaque stability [17, 154]. These various effects are likely to be highly significant in the setting of chronic statin therapy as a primary and secondary prevention measure of coronary heart disease [135].

Although statins are usually well-tolerated, side effects such as myopathy occur in approximately 10 % of patients receiving treatment [155] and a number of patients are statin resistant (estimates of up to 20 %) or intolerant. Thus, new therapies are necessary to reduce the residual disease burden in these patient populations [156].

### RNA-based mechanisms (Fig. 1)

1. miRNA therapeutics

MicroRNAs (miRNA or miR) are short (20–22 nucleotides) non-coding RNAs [157] modulating gene expression further by downregulating the translation of target mRNAs through the inhibition of post-transcriptional events, through transcript degradation or through direct translational suppression [13, 156]. Estimates based on computational approaches currently find more than 60 % of human genes to be targeted by miRNAs, with many of these interactions being highly conserved throughout evolution [158].

In mammals, more than 1000 different *miRNAs* have been described. Among these are cardiac miRNAs, which, as studies indicate, can potentially be modulated by oligonucleotide-based therapies (Table 1) [10, 13, 107, 156, 159–181].

**Table 1:** Mechanisms, clinical relevance, targets and development of microRNA-based therapeutics by companies

	Mechanisms and clinical relevance	Target(s)	Development of microRNA-based therapeutics by companies	References
miR-208	1. Inhibition of miR-208a prevents cardiac remodeling 2. Role in cardiac fibrosis not yet fully identified	p21	x	[10, 107]
miR-33	1. Targets genes involved in HDL metabolism. Preclinical models in which anti-miR-33 was delivered for up to 12 weeks have shown no adverse effects of the approach (assessed by liver enzymes, plasma cytokine levels, blood chemistry panels, blood counts, body weight) 2. Directly target macrophages and cause a regression of atherosclerosis	ABCA1, ABCG1, AMPK alpha, CPT1A, CROT, HADHB, IRS2, NPC1, PRKAA1, SREBP-1	Anti-miR oligonucleotide against miR-33a/b for treating atherosclerosis and dyslipidemia	[156, 160, 179]
miR-146	Pathogenesis and clinical manifestation of atherosclerosis	CD40L, IRAK1, IRAK2, TLR4, TRAF6		[180, 181]
miR-15 family (including miR-15, miR-16, miR-497)	Associated with cell cycle arrest and survival by regulating anti-apoptotic and cell cycle genes	CARM1	Anti-miR towards miR-15 for post-myocardial infarction remodeling of the heart. An 8-mer (nucleotide) directed against the seed region of the miR-15 family: more effective in the derepression of target genes than the previously used LNA-modified 16-mer	[161, 162]
miR-23a, miR-23b, miR-24, miR-195, miR-214	Overexpression of these microRNAs causes hypertrophy in human cardiomyocytes	CDC42 (miR195)	anti-miR towards miR-195 for post-myocardial infarction remodeling of the heart.	[161–163]
	Overexpression of miR-195 in the heart is a sufficient cause for heart failure			
	Transgenic miR-195 mice may develop dilated cardiomyopathy			
miR-133	Overexpression of miR-133 inhibits cardiac hypertrophy	SP1		[163, 164]
miR-34	The response of the heart to stress, including myocardial infarction, leads to an upregulation of miR-34. Involved in cardiac hypertrophy and fibrosis	SIRT1	LNA-modified anti-miR against miR-34a aimed at improving systolic pressure and increasing angiogenesis	[165]
miR-29	miR-29 is implicated in cardiac fibrosis and is downregulated after myocardial infarct and after cardiac injury	LPL (miR-29a)	Development of a pro-miR to target multiple components of the fibrosis pathway	[166]
		DNMT3B (miR-29b)		
miR-21	miR-21 levels in cardiac fibroblasts lead to a decrease in its target mRNA, sprouty-1 (Spry1), a negative regulator of ERK-MAP kinase activity, as well as fibroblast growth factor-2 (FGF2) secretion	BCL-2, PDCD4,	ASO to miR-21 in order to elevate Spry1 expression, to reduce FGF2, and therefore to decrease fibroblast growth	[167–169]
		PPARalpha,		
		PTEN, TPM1, TLR4		
			Anti-miR-21 may help treat a variety of fibrotic conditions, including cardiac fibrosis	
miR-155	miR-155 has been implicated in viral myocarditis. An LNA-anti-miR directed against murine miR-155 reduced myocardial damage during myocarditis	AT1R, ETS-1, MLCK, BCL-2, ETS-1, FADD, HBP1, MAP3K10	x	[13, 170–173]
	The inhibition of endogenous miR-155 has clinical benefit for both cardiac hypertrophy and heart failure			
miR-145	Genetic deletion of miR-145 results in excessive remodeling of the right ventricle and decreasing blood pressure. After vascular injury, the cytoskeleton of smooth muscle cells is modulated by a downregulation of miRNA-145	JAM-A	x	[172]
miR-221, miR-222	Proliferation of smooth muscle cells is partially enhanced by an increase in endogenous miRNA-221 and miRNA-222 levels	c-Kit, eNOS, ETS-1, PAK1, p27, p57, STAT5A		[163]
miR-126	As atherosclerosis develops, the inflammation of vessel walls is enforced by a downregulation of miRNA-126 promoting the expression of VCAM-1 (vascular cell adhesion molecule) and inducing the production of CXCL12 (C-X-C motif chemokine 12), which in turn leads to the recruitment and adhesion of further inflammatory cells	BCL-2, FOXO3, IRS1		[174, 175]
miR-217	When expression of miRNA-217 in atherosclerotic plaques increases, the endothelium disintegrates, which then leads to the inhibition of SIRT1 that causes an acceleration of vascular senescence	SirT1		[176]
miR-1	In developing mouse hearts, the overexpression of miR-1 causes decreased cardiomyocyte proliferation and premature differentiation. Experiments with mice suggest that transient downregulation of miR-1 may prove to be of therapeutic benefit to patients suffering from acute myocardial infarction	MLCK, KLF4, MRTF-A, PIM-1		[13, 177, 178]
	miR-1 negatively regulates key components of calcium signaling pathways and fetal gene activation, making it a vital part of agonist-induced cardiomyocyte hypertrophy in the mouse			

*ABCA1* ATP binding cassette transporter A1, *ABCG1* ATP binding cassette transporter G1, *AMPK*α AMP kinase subunit-α, *AT1R* angiotensin II type 1 receptor, *BCL-2* B-cell lymphoma 2, *CARM1* coactivator-associated arginine methyltransferase 1, *CDC42* cell division control protein 42, *CPT1A* carnitine palmitoyltransferase 1A, *CROT* carnitine O-octaniltransferase, *DNMT3b* DNA methyltransferase 3b, *eNOS* endothelial nitric oxide synthase, *ETS-1* E26 transformation-specific sequence 1, *FADD* Fas-associated death domain-containing protein, *FOXO3* forkhead box O3, *HADHB* hydroxyacyl-CoA-dehydrogenase, *IRAK1* interleukin-1 receptor-associated kinase 1, *IRAK2* interleukin-1 receptor-associated kinase 2, *IRS1* insulin receptor substrate 1, *IRS2* insulin receptor substrate 2, *HBP1* HMG box-transcription protein 1, *JAM-A* junctional adhesion molecule-A, *LPL* Lipoproteinlipase, *MAP3K10* mitogen-activated kinase kinase kinase 10, *MLCK* myosin light chain kinase, *MRTF-A* myocardin-related transcription factor A, *MYL9* myosin light chain 9, *NOX4* NADPH oxidase 4, *NPC1* Niemann-Pick C1, *PAK1* p21/Cdc42/Rac1-activated kinase 1, *PDCD4* programmed cell death 4, *PPAR*α peroxisome proliferator-activated receptor-α, *PRKAA1* protein kinase, AMP-activated, α 1 catalytic subunit, *PTEN* phosphatase and tensin homologue, *SIRT1* sirtuin 1, *SirT1* silent information regulator 1, *SREBP-1* sterol regulatory element-binding protein 1, *STAT5A* signal transducer and activator of transcription 5A, *TLR4* toll-like receptor 4, *TPM1* tropomyosin 1, *TRAF6* TNF receptor-associated factor 6

miRNAs may possibly be used as *diagnostic biomarkers* of, e.g., heart disease [182], as they have been found in the serum and plasma of humans and animals and are highly significant in the pathogenesis of cardiovascular diseases. miRNAs qualifying as biomarkers are circulating miRNA-126 and miRNA-145, which are reduced in the serum of patients with coronary artery disease [183], and miRNA-1, miRNA-133b, and miRNA-499, which are elevated in patient and animal models during acute myocardial infarction [184, 185].

Biological gene networks may be altered by a dysregulation of miRNAs in disease states. miRNA replacement therapy or anti-sense inhibition of miRNAs may aid in restoring gene expression in the cell to its normal state. In the same way, gene networks, such as those controlling key cellular processes like cholesterol efflux, can be targeted by miRNAs, making their characteristic modulation of entire gene pathways instead of single targets a new approach for the treatment of disease [156]. The ability to target single miRNAs and to alter the expression of gene networks provides an exceptional approach to drug development that moves beyond the “one-drug-one-target” mode of treatment.

miRNA therapeutics bear great potential for developing new CVD treatments. Preclinical studies already prove this through early successes with miRNA inhibitors and mimics [156]. As such, a number of RNA-based therapeutics are currently being developed by biotechnological companies. Among these innovations are miRNA (microRNA) sponges, miRNA mimetics, anti-miR oligonucleotides, and anti-sense oligonucleotides (ASOs) [156].

*miRNA sponges* are molecules developed to inhibit intra cellular miRNAs [186] and thus act as competitive inhibitors of the respective miRNA. The sponge binds to the miRNA of interest to prevent the latter from binding to its targets. Difficulties in determining the appropriate dosage may prove to be a disadvantage of this approach. A highly expressed miRNA, for instance, may require a potentially unfeasible dose of sponge to be silenced, whereas an abundance of miRNA target genes would need a much lower dose of sponge to silence the miRNA [156].

*miRNA mimetics* are small, chemically modified double-stranded RNAs mimicking the function of an endogenous miRNA. They are delivered as perfect complementary duplexes to improve RNA-induced silencing complex (RISC) loading of miRNA [187]. Their efficiency can furthermore be enhanced by increasing the affinity for a specific target and reducing other unwanted miRNA effects [188].

*Anti-miR oligonucleotides*, chemically modified to enhance target affinity, stability, and tissue uptake, are a promising approach [189]. Preclinical studies in mice and non-human primates have shown that these compounds rapidly leave the plasma upon systemic delivery. After entering these cells, the anti-miR forms a stable, high-affinity bond with the miRNA, which reduces the availability of the same to bind with the 3′UTR of the miRNA target [156]. Chemically modified anti-miRs have proven to be therapeutically beneficial in mouse models of cardiac dysfunction [156].

*ASOs* are single stranded, short, synthetic 14–22 nt highly specific, complementary oligonucleotides that localize to the nucleus and target a single gene through the interruption of mRNA translation. This in turn is achieved by an RNase H cleavage mechanism that degrades the transcript [190]. Thus, the target mRNA cannot be translated and the level of protein is reduced as it is prevented from reaching the ribosome [191, 192].

*Mipomersen*, a first-in-class ASO inhibitor recently approved by the FDA, inhibits a synthesis of messenger RNA (mRNA) of apolipoprotein B (Apo B) in the liver. Kynamro furthermore reduces LDL-c for the treatment of homozygous familial hypercholesterolemia. These findings enable other oligonucleotide-based therapies and thus bring miRNA therapeutics closer to clinical practice [193].

Another LDL-c lipid-lowering agent is *lomitapide*. Lomitapide and mipomersen both lower LDL-c through the reduced production of hepatic VLDL, which allows them to act independently of an LDL receptor, making them suitable treatment options for patients with homozygous familial hypercholesterolemia.

However, hepatic fat accumulation is intrinsincally linked to the processes connected to lomitapide and mipomersen. The long-term implications of this negative effect are unknown at this time [193]. Lomitapide can also cause gastrointestinal side effects, like diarrhea and nausea, which are, however, manageable by decreasing the medication dose and maintaining a strict low-fat diet. Lomitapide as a cytochrome P450 3A4 inhibitor has potential for drug-drug interactions with other cytochrome P450 3A4 inhibitors and drugs metabolized by cytochrome P450 3A4 [193]. Mipomersen, on the other hand, can lead to injection-site reactions and flu-like symptoms [193].

Kynamro currently has approval for treatment of familial hypercholesterolemia [194–196], a genetic disorder of lipid metabolism characterized by elevated LDL-c, as well as an increased risk of suffering premature coronary heart disease [156].

2. Long non-coding RNAs (lncRNAs)

While research predominantly discusses small non-coding RNAs, such as microRNAs, long non-coding RNAs (lncRNAs) are gaining more prominence as regulators of gene expression. The central role that lncRNAs play in heart development is only slowly being recognized. In addition, understanding the function of these molecules in CVD is even further away [13].

Long non-coding RNAs are a large and diverse class of transcribed RNA molecules, exhibiting a length of more than 200 nucleotides that do not encode proteins and are primarily located in the nucleus [197]. Long non-coding RNAs function either by binding to DNA or RNA in a sequence-specific manner or by binding to proteins [197]. They are transcribed as overlapping sense and anti-sense transcripts of coding DNA regions responsible for the regulation of the transcription of corresponding overlapping mRNA [198]. LncRNAs are functionally distinct from small non-coding RNAs, namely miRNAs, as the latter primarily mediate post-transcriptional repression in the cytoplasm. Certain lncRNAs are precursors for smaller regulatory RNAs such as miRNAs or piRNAs [197]. lncRNAs play a significant role in epigenetic regulation as they, for instance, mediate the activation or repression of target genes through methylation of DNA posttranslational histone modifications [13, 199]. Their expression is developmentally regulated and can be tissue- and cell-type specific [197]. As lncRNAs are believed to bear important regulatory functions, they add yet another layer of complexity to our understanding of genomic regulation [197].

The lncRNA anti-sense non-coding RNA in the INK4 locus (ANRIL) [200–209] modulates atherosclerosis susceptibility at Chr9p21.3 and is overexpressed in human atherosclerotic plaques [201, 210]. ANRIL is furthermore a mediator of epigenetic regulation [13]. The overexpression of ANRIL moreover causes accelerated proliferation and increased adhesion, as well as decreased apoptosis [205], both of which are key mechanisms of atherogenesis [205].

The lncRNAs FOXF1 adjacent non-coding developmental regulatory RNA (*FENDRR*) and *Braveheart* partake in defining the gene transcription program responsible for heart development and cardiomyocyte differentiation, respectively, which indicates they may also be involved in heart failure [211, 212].

## Discussion and limitations

To our knowledge, no epigenetically active agents or drugs targeting histone acetylation and/or methylation have thus far entered clinical trials for CVD, nor have any of the latter been approved by the FDA (US Food and Drug Administration). Data on the potential use of epigenetically active compounds concerning these pathologies are, in fact, merely provisional. The complex relationship between epigenetic regulation and CVD development clearly demands further studies.

Epidemiological and clinical studies have shown that lifestyle modifications including nutritional habits and exercise are first protective measures for reducing the risk of CVD. Nutritional factors in particular are essential for preventing cardiovascular diseases. This comprises both avoiding undesirable food supplements, such as high concentrations of salt and low-density lipoproteins, while simultaneously emphasizing nutritional ingredients with beneficial health effects. Food components such as polyphenols, cocoa, and folic acid are known to affect epigenetic signaling pathways including DNA methylation, whereas it should be kept in mind that excessive application of folic acid significantly increases the risk of carcinoma.

Although clinical practice does not yet use epigenetically active molecules in the therapy of atherosclerosis-related CVD, currently available therapies, such as those using statins to promote epigenetic-based control in CVD prevention through histone modifications, are already moving towards an exploitation of these mechanisms.

While it has been more than 20 years since NO was identified as an endogenous agent produced by the cardiovascular system, until recently attempts to create acceptable therapeutic measures for the modulation of endogenous NO activity or production have not progressed much. NO and its signaling responses possess complex features of chemistry, biochemistry, and molecular biology. The development of targeted therapies for NO delivery or agents that enhance endogenous NO production is intrinsically difficult and the optimal supplemental therapies to significantly increase the positive effects of NO donors or endogenous NO are equally difficult to determine. Thus, all of these issues connected to an epigenetic treatment of CVD require additional clinical study [135].

Non-coding RNAs were found to be important in the pathogenesis of cardiovascular disease and also offer the possibility of operating as diagnostic and prognostic biomarkers [213]. Recent studies—though limited to animal models—have suggested that miRNA inhibition could be an effective therapeutic approach in CVD. Several classes of RNA therapeutics are currently under clinical development by biotechnology companies, including anti-sense oligonucleotides as well as microRNA mimetics and inhibitors.

The use of miRNA and their targets as diagnostic markers or as therapeutics for CVD is promising, but has not yet been realized. As each miRNA may post-transcriptionally regulate 100 different mRNAs, it is difficult to connect any particular miRNA to a specific disease [13, 214]. For this reason, it would be preferable to restrict changes in miRNA levels to diseased cells. While the stable inhibition of target miRNAs in specific cell types cannot be achieved through any methodical means currently available, the future is likely to hold some promising opportunities [13].

Further studies are necessary to improve our understanding of the involvement of lncRNA in regulating gene expression changes underlying heart failure, for example. Through the data won from such studies, it could be possible to develop specific therapeutic strategies for heart failure, for example, on the basis of interference with lncRNA pathways. The role of lncRNAs (e.g., FENDRR, Braveheart) in heart development is now emerging. lncRNA mechanisms may prove successful in preventing and treating different CVD [210].

## Conclusions

Cardiovascular diseases confront mankind with vast and daunting health and financial burdens. Improving preventive and therapeutic measures against them is becoming increasingly necessary. Research efforts should particularly aim at the primary prevention of CVD. There is a promise for enhancing the therapeutic armamentarium against a variety of cardiovascular diseases, particularly as cardiovascular tissues are now being targeted with epigenetic therapies.

## Abbreviations

ANRIL: anti-sense non-coding RNA in the INK4 locus
ASOs: anti-sense oligonucleotides
CHD: coronary heart disease
CVD: cardiovascular disease
DAC: 5-aza-2-deoxycytidinedemethylating agent
DNMT: DNA methyltransferase
eNOS: endothelial nitric oxide synthase
GATA4: GATA binding protein 4
HAT: histone acetyltransferase
HDAC: histone deacetylase
HDL: high-density lipoprotein
LDL: low-density lipoprotein
LNAs: locked nucleic acids
lncRNAs: long non-coding RNAs
miRNA: microRNA
NO: nitric oxide
TSA: trichostatin
